# Field based pilot-scale drinking water distribution system: Simulation of long hydraulic retention times and microbiological mediated monochloramine decay

**DOI:** 10.1016/j.mex.2018.06.015

**Published:** 2018-06-30

**Authors:** Veerdhawal Kulkarni, John Awad, Adam Medlock, Paul Monis, Melody Lau, Barbara Drigo, John van Leeuwen

**Affiliations:** a Natural and Built Environments Research Centre, School of Natural and Built Environments, University of South Australia, South Australia, 5095, Australia; b Future Industries Institute, ITEE, University of South Australia, South Australia, 5095, Australia; c Public Works Department, Faculty of Engineering, Mansoura University, Egypt; d TRILITY Pty Ltd, Adelaide, South Australia, 5000, Australia; e Australian Water Quality Centre, SA Water Corporation, 250 Victoria Square, Adelaide, South Australia, 5000, Australia

**Keywords:** Simulated pilot distribution system, Pilot distribution system, Draw & fill system, Fm test, Microbiological monochloramine decay, Hydraulic retention times, Microflora

## Abstract

Drinking water distribution systems with long hydraulic retention times (HRTs) commonly encounter rapid microbiological-mediated monochloramine decay that results in microbial regrowth and nitrification with reduction in alkalinity. In this paper, we report the design and operation of a field-based pilot-scale distribution system (PDS) operated at flows that simulate long HRTs (∼10 days) to promote rapid microbiological monochloramine decay over long periods. The PDS is designed to enable the testing of chemical treatment for the control of nitrification and monochloramine decay. The PDS has two identical cylindrical polyethylene tanks (DS_1_ & DS_2_), each of 1 m diameter and 1.8 m height (∼1 kL) holding 900 m of polyethylene (PE) tubing with sampling points every 300 m intervals. Microbial mediated decay (determined by the Fm test) of monochloramine occurred as treated (alum coagulated and flocculated) and chloraminated water passed through the DSs. In this manuscript we report:

• An inexpensive, flexible and compact system that can be readily set-up at a full-scale water treatment plant, requiring minimal supervision for operation.
• A ‘draw & fill’ system for achieving control on HRT’s through the pipes.

## Graphical abstract

**Figure:**
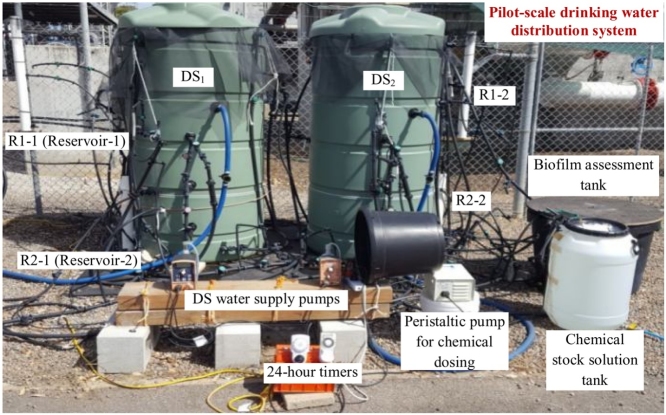

**Specifications Table**

**Table:** 

Subject area	***Engineering***
More specific subject area	***Water Engineering***
Method name	***Simulated pilot distribution system***

## Details of materials used for the pilot-scale drinking water distribution system

• 2 × PE tanks (1.8 m height, 1 m diameter, ∼1 kL capacity) with lids and 2 × 1 m^2^ fine wire mesh (as additional covering);
• PE tubing (900 m × 13 mm diameter/tank);
• Additional 13 mm and 19 mm PE pipes for external connections;
• 13 mm and 19 mm PE-connectors, taps, T-sections, pipe clips and 90° fittings;
• 19 mm to 13 mm PE reducers;
• 2 × float ball valves;
• 2 × mesh cartridge filter (300 μm);
• 2 × low strength peristaltic pumps (ProMinent, Models: gamma/L and Beta/4);
• 2 × 24-h power switch timers;
• Electrical cable and heavy-duty safety power board;
• 200 L tank (for biofilm assessment tank, BAT);
• 4 × ∼100 cm PVC pipes (77 mm internal diameter, for draw and fill of water supplies);
• Nylon ropes;
• Covers (plastic containers) for the pumps and power board;
• 70-L chemical stock solution PE tank;
• Masterflex L/S (77800-50) Easy Load Pump for chemical dosing;
• L/S^®^ tubing- 10 m spare tubing;
• Small and large cable ties

## Method details

### Pilot distribution system (PDS)

#### Installation phase

**Fig. 1:**
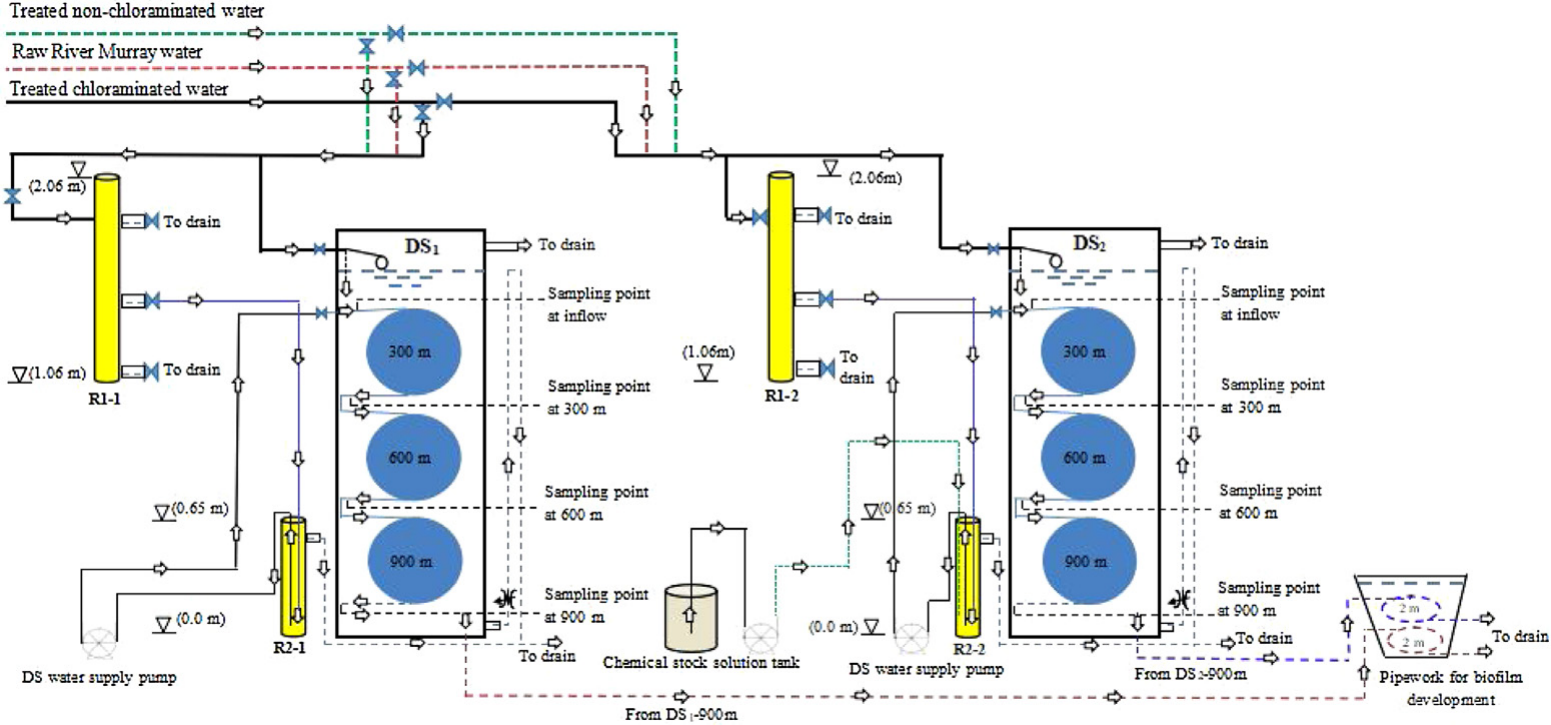
Schematic diagram of the pilot-scale distribution system at Tailem Bend, South Australia.

The pilot distribution system (PDS) was set up at the Tailem Bend Drinking Water Treatment Plant (TBWTP), Tailem Bend, South Australia (35.253 °S, 139.456 °E). The water treatment process (capacity of 28 ML/d) comprises coagulation & flocculation, tube settler clarification, dual media rapid gravity filters, and disinfection by UV (low pressure mercury lamps) and monochloramine [1]. Powdered activated carbon is periodically used and the treated water is fluoridated prior to distribution. The PDS was constructed to simulate a full-scale chloraminated drinking water distribution system and to enable control of water conditions that would allow the study of microbially-mediated and chemically-based monochloramine (NH_2_Cl) decays. The PDS (Fig. 1) consists of two identical cylindrical PE tanks (DS_1_ and DS_2_) of 1 m diameter and 1.8 m height (∼1 kL capacity each). Each tank consists of 3 bundles of PE tubing (13 mm x 300 m each) connected in series (Fig. 1) with supporting connections and sampling points at the inflow, 300 m, 600 m and 900 m. Polymer-based materials (e.g., PVC and PE) are easier to handle and install and consequently are becoming increasingly popular [2]. The water holding capacity of the 900 m pipes is ∼ 120 L. The PE tubing size (13 mm internal diameter) used in the DSs provides comparatively high surface area to bulk water volume, with the ratio of circumference to cross sectional area being ∼0.31 compared to full-scale distribution systems (e.g. for a 100-mm pipe, the ratio is ∼0.04). This was designed to promote biofilm growth and microflora proliferation in the DSs.

**Fig. 2:**
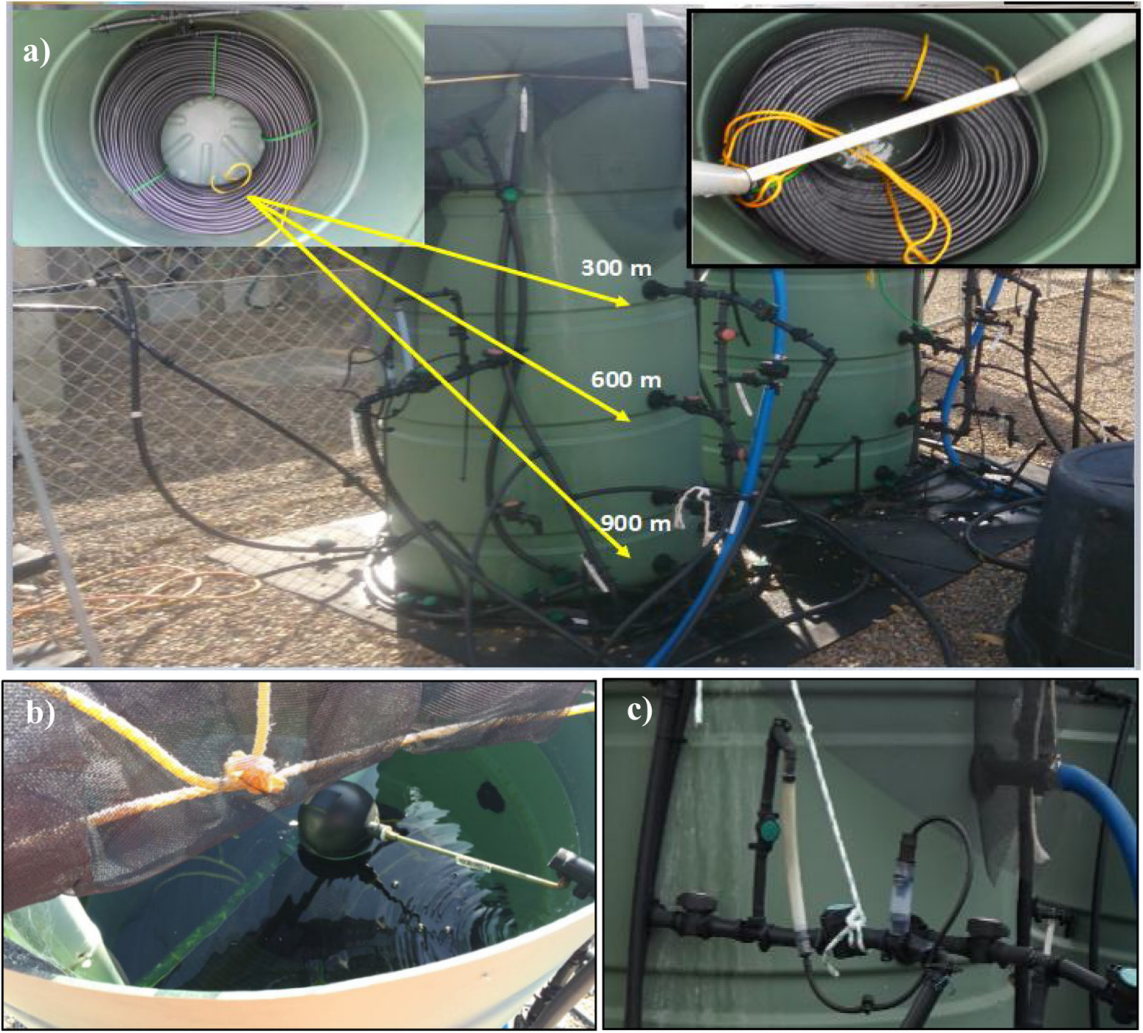
a) Sampling points on one of the DS’s (DS_1_). Top left photo shows one of the 300 m polyethylene bundles connected to the bottom ‘T’ section of the tank. Top right photo shows a PVC pipe arrangement to restrain the PE tubing inside the tanks. b) Float ball valve controls the bulk inflow into the DS. c) Pipework configuration to allow measurement of the flow through the DS.

These tubing bundles remain immersed in treated water sourced from TBWTP that continually flows at a low flow rate through the DSs externally to the tubing in order to stabilise the temperatures in and around the pipes. Without this, the water inside the pipes would experience rapid changes in daily temperatures and might result in physical damage (cracks etc.) to the pipework. The entire tubing is held in position with PVC pipes (see Fig. 2, top right corner) to restrain the tubing from floating inside the tanks, that otherwise might lead to breakage of the internal connections, due to stress from the floating movement of the tubing. This support structure comprises two PVC pipes (50 mm) fixed to the sides of the tank with another pipe inserted horizontally into these pipes at the bottom immediately on top of the bundles to maintain a fixed distance between the vertical pipes and thereby providing stability to this structure (the structure is restrained by cable ties to the rim of the tank).

The pipework was designed to provide controllable hydraulic retention times (HRTs) that simulated long HRTs of full scale distribution systems. The outflow is measured through a pipework configuration shown in Fig. 2c. This includes diverting the outflow line at the bottom and raising it at exactly same level of the main feeding line to achieve the same head pressure. This provided accuracy and flexibility in measuring the flows (using a graduated cylinder). The flow is measured as ml/min and then the HRT for each tank is calculated by using Eq. (1). The bulk water flow (external of the 13 mm pipework) from the tanks and outflows from the 13 mm pipework at 900 m, drains continuously into the TBWTP’s main drain. Pipework flow rates are low, at about 12 L/d for 10 days HRT.

HRT (days)  =  (V/Q) (1)

where, V is the water holding capacity (L) of 900 m pipework (∼120 L), Q is the outflow (L/d) at 900 m pipework

#### Commissioning phase

The PDS was supplied with three optional water sources, raw water (sourced from the River Murray), treated non-chloraminated water and treated chloraminated water. The design allows inflow of any of the three water sources and selected mixtures. These waters can be supplied either under gravity to the PDS or using a ‘draw & fill’ system (using DS water supply pumps). Small low-density PE inline filters (diameter: 13 mm) with coarse mesh cartridge filter (300 μm) were installed on the feeding lines to the DSs. This was done to filter coarse debris.

During the commissioning phase, the main aim was to develop microflora (and biofilm) inside the pipes to simulate conditions in a full-scale water distribution system and consequently to have consistent microbially-mediated monochloramine decay over a long period of time. Both DSs were fed with a mix of raw (80%) and treated (20%) chloraminated water under gravity over a period of 12 weeks. This was done to introduce source water microflora into the pipework with exposure to lower levels of chloramine (0.8–1.0 mg/L) than doses applied at the full-scale WTP, i.e. to promote microflora development that could rapidly decay monochloramine. The 900 m of 13 mm PE pipes provided a relatively large surface area (to bulk water) for the formation of biofilm. Shaw et al. [3] reported that bacterial re-population occurred in post-disinfected water in the distribution system that resembled microflora in the raw source water. The potential option of seeding the pilot plant with selected microbial strains (e.g., AOB) was not chosen and instead, seeding occurred by microflora in waters sourced from the full-scale water treatment plant. Although seeding of particular strains is feasible, the microbial populations within the pilot plant would be continually impacted by the continuous inflows of supply waters and constituent microbial communities sourced from the full-scale water treatment plant.

#### Operational phase

During the operational phase, water supply to both DSs was switched to 100% treated chloraminated water. The aim was to study microbially-mediated monochloramine decay under different flow rates and water conditions. Treated chloraminated water flows under pressure from the full-scale treatment plant into a small holding tank (R1: PVC pipe with 77 mm diameter, length: 1 m), and subsequently under gravity and valve control to a second holding tank (R2: PVC pipe of diameter 77 mm and length, 65 cm), Fig. 1. With consistent head pressure from R1 to R2, gravity feed water flow rate to R2 was able to be controlled consistently. This was done to minimize the effect of variable WTP flow pressures on water flows to the PDS, i.e. flows to R1 were always at rates that ensured the tank was full to overflow, thus maintaining consistent head pressure from R1 to R2.

Flow rate to the R2 was maintained to achieve a consistent overflow that ensured R2 being continually full for supply to the DS. Overflows are connected to the drain of the PDS. Water from the R2 is pumped directly into the DSs using diaphragm pumps (ProMinent, Models: Gamma/L; Beta/4) to achieve target HRTs. In the first experiment; these were operated for a fixed period of 2.5 h continuously/day to simulate dead-end sections of drinking water distribution networks. This was done to promote the development of microflora capable of causing rapid monochloramine decay simulating a scenario of stagnant water conditions in full-scale distribution systems. With application of the water supply pumps, consistent HRT’s were able to be achieved. Also, water supply pumps have been operated for set time periods distributed over 24 h. Based on the operation of the water supply pumps, the DSs can be used to simulate the transmission mains (water flow continuously at high flow rates and low HRTs) or to simulate the dead-end sections of drinking water distribution networks (water flows for a fixed period of hours per day to represent periodic stagnant water conditions).

#### Chemical dosing system

The PDS is designed to enable the testing of chemical treatments for the control of nitrification and monochloramine decay. The chemical dosing system comprises a 70 L plastic tank as chemical stock solution reservoir, a Masterflex peristaltic pump (Model No. 77800-50) with LS 16 tubing (diameter: 3 mm) and timer (with 15-min. interval settings). The feeding line from the chemical dosing pump is inserted into R2 (Fig. 1) of DS_2_ and is positioned to the bottom to the level of the incoming treated water line from R1 for efficient mixing of treated water and chemical before entering into the DS_2_. The chemical dosing into R2 is adjusted to commence 45-mins before the start of DS water supply pump. This would provide sufficient mixing time of the test chemical with treated monochloraminated water in R2 before entering into DS_2_ distribution system. Factors such as target dose of chemical, concentration and volume of the chemical stock solution along with dimensions of the holding tanks, water flow rates from R1 to R2 and controlled flow from R2 to DS, dictate the chemical supply rate into R2, and timing of the pump operation.

#### Pipework for biofilm measurement

**Fig. 3:**
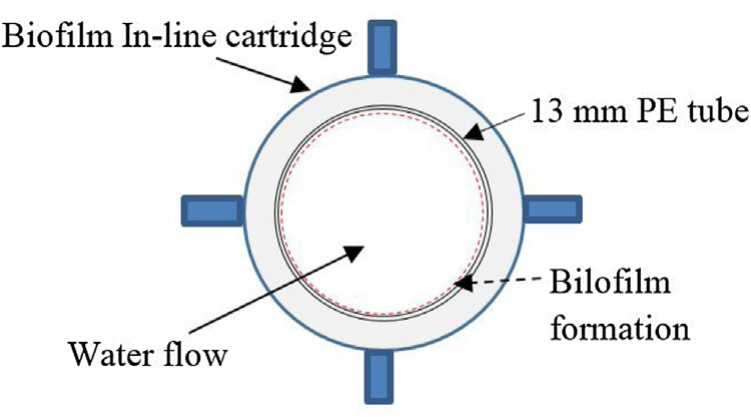
Cross section view of the 13 mm PE pipe piece inserted into cartridges.

In drinking water distribution systems, one of the major problems caused by low chloramine residuals is microbial regrowth [4]. Nitrification is also reported to be a common problem associated with chloramination. It has been reported that ammonia oxidizing bacteria (AOB) and nitrite oxidizing bacteria (NOB) are the major contributors towards nitrification that causes monochloramine decay [5]. Regan et al. [5] reported that *Nitrosomonas (Nm. oligotropha* cluster) and *Nitrospira* were the main AOB and NOB, respectively, of a chloraminated distribution system they studied. To enable the investigation of microflora/biofilm of the PDS, an extension was installed adjacent to the 1 kL PE tanks (Fig. 1). For this, the outflows from DS_1_ and DS_2_ are diverted into a 200 L PE tank (biofilm assessment tank [BAT], see Fig. 1). The length of the pipes inside the BAT is around 2 m and these pipes remain immersed in WTP supply water at all times. These pipes are fitted with in-line cartridges (pipework material) for biofilm formation and sampling. Two small pieces of PE pipe, (length: 10 cm/piece) were placed in each cartridge (Fig. 3) to provide a substrate for biofilm growth. Initially, outflow from DS_2_ was passed through both pipelines of the BAT for logistical purposes and subsequently flow from each DS was connected to separate feeding lines of the BAT.

### Water quality analysis

#### Chemical and physical analysis

**Fig. 4:**
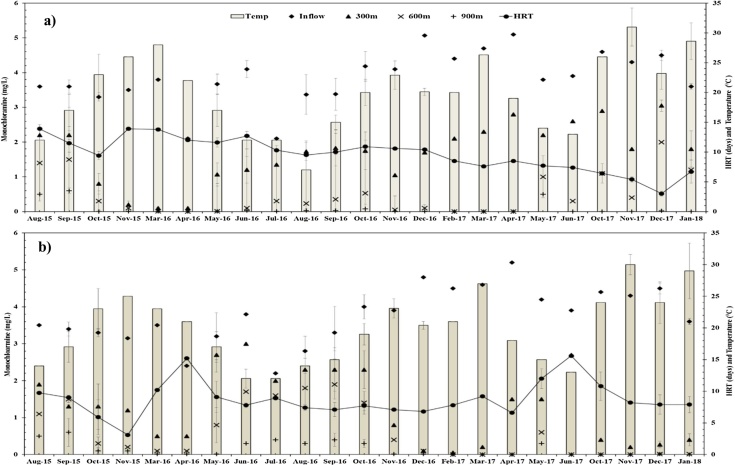
Average NH_2_Cl decay as the water passes through the entire DS_1_ (a) and DS_2_ (b).

On an average, water samples were collected three times per month from the PDS at inflow, 300 m, 600 m and 900 m (outflow) sampling points of the DS. Free and total chlorine concentrations (mg/L) were measured immediately after sample collection using a DR 2800™ Spectrophotometer (HACH, Australia), at the TBWTP. Monochloramine concentration was calculated as the difference between total and free chlorine concentrations. Water sample analyses using the DPD-Ferrous Titrimetric method Standard Method no. 4500-Cl F [6] showed 91% (S.D. 7.6%, n = 12, chloramine range 1.5 to 4.6 mg/L) of total chloramine was monochloramine and these values were similar to those measured by using DR 2800™ Spectrophotometer (HACH, Australia). The pH values were immediately determined using a portable pH meter (WP-91, TPS Instruments), and water temperatures were recorded using digital temperature thermometer (TR-10- Elitech) at TBWTP. Average monthly pH levels for the PDS during the study period have been previously reported [7] while average monthly water temperatures are presented in Fig. 4. Nitrate and nitrite concentrations were measured using Spectroquant NOVA 60 (MERCK, Australia) and cell test nos. 109713 and 114547 respectively for waters collected from DS_1_.

#### Microbial factors

**The microbial decay factor (Fm) test** was used, as described by Sathasivan et al. [8]. Fm is the ratio between the microbial decay coefficient (k_m_) and chemical decay coefficient (k_c_) of a first order decay function. A first-order decay function (Eq. (2)) was applied to model the decay of monochloramine. Two monochloramine bulk decay coefficients for each individual sample (k_t_ for unfiltered sample and k_c_ for filtered sample) were determined using Tablecurve^©^ 2D Curve Fitting Software. Using these decay coefficients, the k_m_ value (k_t_ - k_c_) and Fm value (k_m_/k_c_) for each individual sample were calculated. Fm test conditions (e.g., pH level, water temperature, chlorine/ammonia ratio), chemicals preparation and monochloramine residual measurement method and other relevant information for the Fm method have been previously reported [7].

C_NH_2_Cl_ = C_NH_2_Clt = 0_ × exp ^(−k × t)^ (2)

##### Heterotrophic plate count (HPC)

Water samples were collected from DS_1_ at the inflow, 300 m, 600 m and 900 m for determination of heterotrophic bacteria concentrations using HPC (in June 2018). Incubation was performed at 22 °C for 72 h as described in the Australian Standard AS/NZS 4276.3.1. Following incubation, colonies were counted using an illuminated magnified (× 2.5) colony counter.

##### Microflora DNA extraction, quantification and quality

Water samples were collected in 500 mL RNAse/DNAse free bottles at the PDS outflow (in May 2017) and were dosed with sodium thiosulfate (18 mg/L) immediately on collection to neutralize residual chlorine. Samples were then vacuum-filtered through 47 mm diameter, sterile plain cellulose nitrate membranes, 0.1 μm pore size (Whatman, GE Healthcare Life Science, NSW, Australia) immediately on arrival at the laboratory. DNA was extracted from each filter using DNeasy PowerWater kits (Qiagen, NSW, Australia) according to the manufacturer’s instructions. For biofilm analyses, portions (@ 4 cm) of the PE pipes from the biofilm collectors were cut (in February 2018) and placed in sterile Nunc 50 mL conical centrifuge sterile tubes and transported to the laboratory on ice. Biofilm on the duplicate pipe pieces was detached by vigorously scraping the surface with RNAse/DNAse free, sterile and antistatic spatulas (Corning, Austin, TX, USA) until complete removal. Approximately 0.20 *g* of biofilm material was recovered from each sample and placed into a 2 mL collection tube. Biofilm material was then suspended in 350 μL of cell lysis solution (DNeasy PowerSoil™ DNA isolation kit; Qiagen, NSW, Australia) and incubated at 65 °C for 5 min. The solution was vortexed (30 s), sonicated (30 s) and vortexed with the FastPrep-24 Classic (Mp Biomedicals, Santa Ana, California, USA) for 4 cycles of 60 s each. The tubes were centrifuged at 13,000 x g for 1 min at room temperature and the supernatant collected in a sterile 2 mL collection tube. Genomic DNA was then isolated from the supernatant using the DNeasy PowerSoil™ DNA isolation kit according to the manufacturer’s specifications (Qiagen, NSW, Australia). DNA was eluted in 100 μL of RNAse and DNAse free water and stored at -20 °C until further analyses.

The DNA extracts from the water filters and biofilms were quality checked by 1.5% agarose gel electrophoresis. The DNA yield was measured using NanoDrop, 2000 Spectrophotometer (Thermo Fisher Scientific, Australia) and the A260/A280 ratio was on average 1.9. The DNA concentration in extracts ranged between 45–50 ng/μL for the water filters and 95–100 ng/μL for the biofilm extracts. To assess the presence of PCR inhibitors in the DNA extracts, bacterial 16S rDNA copy numbers were quantified using the QX200™ ddPCR™ EvaGreen Supermix (Bio-Rad, Australia) on the Bio-Rad CFX96™ Real-Time system (Bio-Rad Laboratories, Hercules, CA, USA). Quantification of bacterial SSU ribosomal DNA was carried out using 519f/Ba907 r by Lueders et al. [9]. Each reaction mixture (25 μL) consisted of 12.5 μL QX200™ ddPCR™ EvaGreen Supermix (Bio-Rad, Australia), 2.5 μL of water, 2.5 μL (10 μM) of each primer, 2.5 μL of BSA and 5.0 μL of 10 times diluted DNA. Quantitative PCR (qPCR) followed a standard two-step protocol consisting of 15 min at 95 °C, followed by 40 cycles of 95 °C for 60 s, 52 °C for 60 s and 72 °C for 60 s. *Escherichia coli* reference strains were grown in Luria Bertani (LB) broth at 37 °C with shaking (100 rpm). *E. coli* growth was monitored by determining the optical density at 600 nm (OD600). For establishing the number of viable *E. coli*, samples were serially diluted in LB broth and 50 μL was spread plated on LB agar plates in replicates. Plates were incubated at 37 °C for 24 h. For calculation of the CFU per millilitre, only dilutions showing between 20 and 300 colonies were used. *E. coli* DNA extraction was performed with the DNeasy Blood and Tissue kit, as recommended by the producer (QIAGEN, NSW, Australia). For DNA extractions, the lysis was performed with 100 μL of lysozyme (500 μg/mL) for 5 min at room temperature. The numbers of copies of the qPCR standards were calculated by assuming average molecular masses of 340 Da for 1 nucleotide of single-stranded DNA. The calculation was done with the following equation: copies per nanogram = (n × mw)/(NL × 10 − 9), where n is the length of the standard in base pairs or nucleotides, mw is the molecular weight per bp or nucleotide, and NL is the Avogadro constant (6.02 × 10^23^ molecules per mol). The biofilm copy number was determined from the standard curve and subsequently standardized to copy numbers per gram of biofilm. In all runs, standard curves and the amplification efficiency (Eff) were calculated using the software manufactured by Bio-Rad and using the following Eq. (3):

Eff = 10^(−1/slope)^ – 1 (3)

The efficiency of the different real-time PCRs ranged from 95 to 100% (−3.64 > slope > −4.31). Secondary structures were not encountered in any of the runs. The threshold of each single run was placed above any baseline activity and within the exponential increase phase. The cycle thresholds (C_T_) were determined by a mathematical analysis of the resulting curve using the software manufactured by Bio-Rad. The C_T_ values of the non-template controls (NTC) were always around 40, indicating no amplification and internal positive controls (*E. coli* ATCC 25922) were around 25. Melting curves were determined for qPCR products to confirm product integrity and asses the presence of inhibitors, including the presence of primer-dimers. Among the different qPCR coefficients, particular attention was given to the R^2^ coefficient which was used to analyse the standard curves obtained by linear regression analysis. For each run, the R^2^ was ≥0.99 values between zero and −1 a negative correlation and between zero and +1 for a positive correlation. Most of the samples, and all standards, were assessed in at least two different runs to confirm the reproducibility of the quantification and all the samples ranged between 10^5^ to 10^6^ copies number/μL and were free of PCR inhibitors.

##### Quantitative PCRs Method

The abundance of total bacteria and archaea, ammonia oxidizing archaea/bacteria (AOA/AOB) and nitrite oxidizing bacteria (NOB) were quantified using qPCR. Samples were analysed in duplicate in parallel with a standard ranging from 5-5 × 10^9^ copies/reaction. Table 1 shows the details of primers and PCR condition. Two DNA polymerases: GoTaq (Promega, Australia) and Faststart (Roche, Australia) were used in this study. The total volume of the Go Taq PCR mixture was 25 μL and it contained 2 μL of DNA, 0.2 μM of each primer, 0.2 μM of dNTP, 2.5 mM MgCl_2_, 3.34 μM SYTO^®^ 9 (Thermofisher, Australia) 1 x GoTaq buffer, 1U of Gotaq DNA polymerase. The total volume of the FastStart PCR mixture was 20 μL and it contained 2 μL of DNA, 0.5 μM of each primer, 3.34 μM SYTO^®^9 and 1 x FastStart Essential Probe Master. The initial denaturation was at 95 °C; 3 min for GoTaq and 10 min for FastStart. A quantification analysis was performed using LightCyler^®^96 software to determine the concentration of targeted genes in samples.

**Table 1:** Sequences of different real time PCR primers.

Specificity	Target Gene	Primer sequence (5'-3')	Amplicon (bp)	DNA Polymerase	PCR condition (^o^C/second)	Reference
Domain Bacteria	16S *rRNA*	F: AAACTCAAAGGAATTGACGGGG		GoTaq	40 cycles 95/20, 60/20, 72/30	Lane [10]
		R: GGGTTGCGCTCGTTGC	193			
Domain Archaea	16S *rRNA*	F: TAAAGGAATTGGCGGGGGAG		GoTaq	50 cycles 95/20, 56/20, 72/30	Lueders and Friedrich [11]
		R: GACGGGCGGTGTGTRCA	479			
*Nitrospira lineages*	*nxrB*	F: TACATGTGGTGGAACA		GoTaq	45 cycles 95/20, 57/20, 72/30	Vanparys et al. [12]
		R: CGGTTCTGGTCRATCA	380/485			
*Nitrobacter-like lineages*	*nxrB*	F: ACGTGGAGAGACCAAGCCGGG		GoTaq	45 cycles 95/20, 66/20, 72/30, 87/30	Pester et al. [13]
		R: CCGTGCTGTTGAYCTCGTTGA	380			
ß- proteobacterial ammonia oxidizer	*amoA*	F: GGGGHTTYTACTGGTGGT		FastStart	40 cycles 95/10, 55/10, 72/30	Rotthauwe et al. [14]
		R: CCCCTCKGSAAAGCCTTCTTC	450			
Ammonia oxidizing marine archaeon	*amoA*	F: ATGGTCTTGCTWAGACG		FastStart	45 cycles 95/10, 52/10, 72/30	Zhang et al. [15]
		R: GCCATCCATCTGTATGTCCA	593			

## Method validation

With the PDS system as described, rapid monochloramine decay occurred in both DSs. Average monthly monochloramine concentrations in water samples collected at the inflow, 300 m, 600 m and 900 m (outflow), HRT’s and temperature over the study period for both the DSs are presented in Fig. 4. Over the study period, mean values at inflow were DS_1_: 3.86 ± 0.7 mg/L, DS_2_: 3.76 ± 0.7 mg/L, at 300 m DS_1_: 1.69 ± 0.8 mg/L, DS_2_: 1.28 ± 0.9 mg/L, at 600 m DS_1_: 0.48 ± 0.6 mg/L, DS_2_: 0.59 ± 0.7 mg/L and at 900 m DS_1_: 0.08 ± 0.2 mg/L, DS_2_: 0.15 ± 0.2 mg/L. The differences in residual monochloramine levels between the two DSs are attributed, in part, to controlled variation in HRTs that were applied in trials to test for impacts on monochloramine decay. The mean HRT value for DS_1_ was 9.6 ± 2.8 days and for DS_2_ 8.7 ± 2.7 days. Average monthly water temperatures ranged between 12 and 31 °C for both the DSs as shown in Fig. 4.

Consistent with the residual monochloramine levels (measured at the same time of the HPC samples), the highest HPC value was found for water sample collected at 900 m (1300 cfu/mL at 0.07 mg/L of NH_2_Cl), followed by water collected at 600 m (23 cfu/mL at 0.6 mg/L of NH_2_Cl). Under the standard HPC test applied, heterotrophic bacteria were found to be absent in waters collected at 300 m and at inflow (300 m: 0 cfu/mL at 2.1 mg/L of NH_2_Cl; inflow: 0 cfu/mL at 4.2 mg/L of NH_2_Cl).

During the study period, nitrate and nitrite concentrations at 300 m, 600 m and 900 m were measured. Average nitrite concentrations were found to be higher at 600 m (0.5 ± 0.02 mg/L) compared with 300 m, < 0.03 mg/L (measured 0.01 mg/L) and 900 m (0.04 ± 0.03 mg/L). Average nitrate concentrations were higher in 900 m (0.8 ± 0.1 mg/L) compared with 600 m (0.4 ± 0.1 mg/L) and 300 m (0.3 ± 0.1 mg/L).

To examine the nature of monochloramine decay, water samples were collected from the DSs (inflow, 300 m, 600 m and 900 m) and the microbial decay factor (Fm) test was used as described by Sathasivan et al. [8]. Regardless of the season/temperature effect, higher Fm values were found in the waters collected from the entire PDS than inflow waters (treated water from TBWTP). The highest values of Fm were found for water samples collected after 900 m (DS_1_: 1.6 ± 1.1, DS_2_: 2.5 ± 2.4) of pipes followed by waters collected after 600 m (DS_1_: 1.2, DS_2_: 0.9 ± 0.6), then waters collected after 300 m (DS_1_: 0.2 ± 0.2, DS_2_: 0.9 ± 1.2) and inflow water samples (DS_1_: 0.1 ± 0.1, DS_2_: 0.2 ± 0.2). These data show an increase in microbiologically-assisted monochloramine decay in waters as it passed through the DSs.

Water samples were also collected from the DSs outflow for detection of bacteria known to be associated with the decay of monochloramine. Small pieces of PE pipe material were taken for biofilm analysis. Average counts for domain bacteria, domain archaea, nitrifying bacterial species (i.e. AOB, NB, NS) and AOA present in water and biofilm samples in both DS outflow samples are shown in Fig. 6. Compared to other nitrifying bacterial species, Nitrospira (NS) was found to have the highest abundance while the least detection was for Nitrobacter (NB). Shaw et al. [3] reported that regrowth of bacteria occurred further down in DSs, and for samples collected from sites that had experienced nitrification, nitrifying bacterial species such as NS were detected. The relative abundance was calculated based on the number of target copies detected divided by the total number of bacteria/archaea present. The average of the relative abundances of AOA and NS were found to be higher in the biofilm samples (AOA: 1.3%; NS: 4.4%) compared to that detected in the water samples (0.3% and 2.7%). Low abundances of AOB were detected in both water (0.04%) and biofilm (0.1%) samples, while the abundance of NB was found to be negligible.

**Fig. 5:**
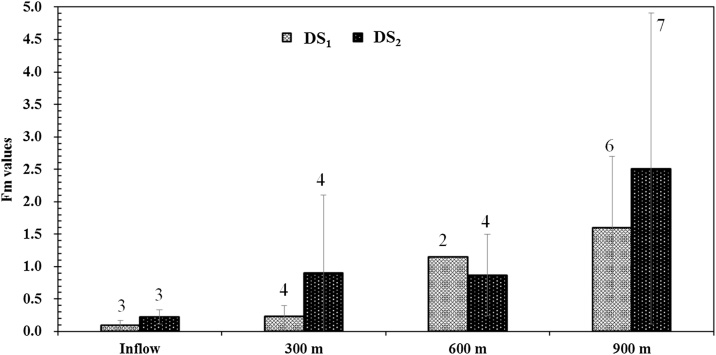
Average Fm values for waters collected from DS_1_ and DS_2_. Values above bars represent the number of samples.

In general, key factors affecting biofilm formation in the distribution system include water characteristics and operational conditions (i.e. organic and inorganic matter, trace elements, temperature fluctuations, pH levels, flow-rate variations) [2]. The PDS was continuously supplied with conventionally treated and chloraminated water from a full-scale WTP (Tailem Bend), where the water quality to the pilot plant is the same as the inflow to the full scale distribution system. Moradi et al. [1] reported that the HRTs of the full-scale distribution system of Tailem Bend are between 7 and 14 days and the average water temperature ranges between 15 and 40 °C. The PDS was operated at long HRTs (∼10 days) and the water temperature inside the pipe network was found to be between 12 and 31 °C. Further, Fm value for water collected from a point of use tap at Raukkan, a location supplied by the Tailem Bend DS (a site that had experienced nitrification- NO_x_ = 1.16 mg/L; HRT ∼7 days) was found to be much higher compared to that in the treated waters from TBWTP (2.7 for the Raukkan customer tap and 0.7 for treated waters from TBWTP). The PDS follows the same trend with higher Fm values for waters collected from the entire PDS than inflow waters (treated waters from TBWTP), Fig. 5. Data acquired by the managing water utility include pH level (7.9) for water collected from the Raukkan customer tap, which is lower than the treated water collected from the TBWTP (8.4). The pH levels measured in waters from the PDS followed the same trend with values lower in water collected from the PDS (300 m: 7.8 ± 0.3; 600 m: 7.7 ± 0.3; 900 m (outflow waters): 7.5 ± 0.2 than that collected from TBWTP (8.4 ± 0.2). This can be attributed to either greater exposure to microbiological activities or progressively more nitrification as the water moves through the PDS**.** Wilczak et al. [16] and Bal Krishna et al. [17] reported that pH (in a laboratory scale reactor system) decreased as a result of increase in AOB activities. Wahman and Pressman [18] and Sung et al. [19] also reported that reduction of pH in drinking water distribution systems is a direct impact of the nitrification process.

**Fig. 6:**
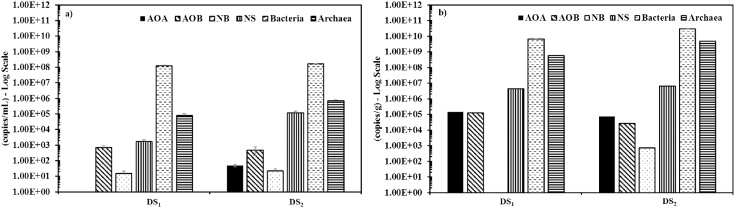
Average measured nitrifying bacterial species presented in (a) water samples and (b) biofilm collected from the DSs outflows.

## Additional information

Chemical disinfection is used in the supply of drinking water to inactivate or kill pathogens found in source waters, such as rivers and reservoirs. Chemical disinfection is applied with the aim to maintain residual concentrations in potable water distribution systems through to the point of supply to consumers to ensure safe water provision. Chemical disinfectants include chlorine, ozone, chlorine dioxide and monochloramine [20]. In Australia, monochloramine is used where drinking water supply is over long distances i.e. distribution systems that have long HRTs due to its general high stability and effectiveness in providing protection from pathogenic micro-organisms such as the amoeba*, Naegleria fowleri*. Although the overall rate of chloramine decay by auto-decomposition is generally much lower compared with the rate of chlorine decay [21], monochloramine will nonetheless degrade over time. According to the Australian Drinking Water Guidelines [22], the average municipal water system maintains a residual monochloramine concentration at around 1.5 mg/L. At the TBWTP, initial monochloramine levels in treated water were ∼ 4.0 mg/L with the Cl_2_/NH_3_–N ratio of 4.5:1 [1].

Rapid biologically-mediated monochloramine decay in drinking water distribution systems occur generally in systems with long HRTs and remain a significant problem for the water industry. Consequently, there is a need for robust, inexpensive, reliable and easily assembled in-field pilot-scale distribution systems, in order to study the effects of operational conditions and water quality on monochloramine, and other disinfectant decays.

A few lab-scale reactor systems and pilot-scale distribution systems have been used to study chloramine decay rates under controlled conditions. Krishna and Sathasivan [23], Krishna et al. [24] and Sarker et al. [25], used five lab-scale HDPE reactor (25 L capacity-each) seeded with specific microorganisms to examine chloramine decay in mildly and severely nitrified waters (under different nitrification scenarios) at temperature ranging between 20 & 23 °C and HRTs ranging between 19 & 22 h. Sawade et al. [26] also used three 20 L batch reactors to investigate the metabolism of ammonia by indigenous bacteria present in filtered water from a water treatment plant. In contrast, the field-based PDS presented here consists of 900 m of PE pipe network (in each DS) with total water holding capacity of 120 L. The PDS can be located at a full-scale WTP, where environmental conditions (i.e. fluctuating ambient source water temperatures, fluctuating organic compounds and concentrations) are similar to the actual network conditions enabling simulation of chloramine decay through the pilot plant DSs.

Harrington et al. [27,28] and Yang et al. [29] operated a laboratory-based two-stage pilot distribution system. However, they used HDPE tanks (7.6 L and 22.8 L) as mains to recycle the water (connected to a PVC pipe loop: 1.3 cm in diameter and 7.6 m in length) and to provide maximum HRTs of 1 and 3 days, respectively. Regan et al. [30] also used two tanks in series to provide maximum HRTs of 1 and 4 days. In another study, Frias et al. [31] used a 200 m long simulation distribution system (1.5 cm diameter PE pipes) with HRT of less than a day (i.e. 10 h). In contrast, the PDS described here was developed to enable long-term investigation of factors affecting monochloramine decay and residuals in simulated distribution systems with long HRTs (∼10 days), and with temperature flux moderation. This PDS, consisting of 900 m of PE pipework (in each DS), enables long HRTs to be achieved without any need to recycle water to feed water to the pilot plant. In another study, Imran et al. [32] developed PDSs consisting of hybrid pipes (PVC, unlined iron, lined iron and galvanized iron) of variable lengths (12–27 m) and diameters (50–152 mm). These were used to examine the effects on monochloramine residuals at two pre-determined HRTs of 2 and 5 days. However, this system was constructed in 8 months, was limited to the study location (Tampa Bay region, Florida, USA) and would be costly to replicate. Although our PDS operates with long HRTs, the system is a highly compact design, constructed from inexpensive and readily available PE components including standard rainwater tanks, 13 mm diameter tubing and standard irrigation parts. The system is flexible (can be easily modified), highly robust and is readily set-up in the field at a full-scale water treatment plant. Once operational it requires minimal supervision. The system can be readily decommissioned, transported and installed to other test sites. This in-field PDS has been operational since 2015 and water samples collected from both the DSs have demonstrated consistently rapid microbiological mediated monochloramine decay throughout the pipework, enabling a range of investigations to be undertaken.

The PDS has been developed with an aim to broaden the understanding of various microbiological, physical and chemical factors and their dynamics with chloramine levels in drinking water and can be used to examine the potential effectiveness of treatment strategies for control of nitrification, monochloramine decay and DBPs formation. The design includes capacity for biofilm development and isolation arrangement for biofilm analyses using in-line PE cartridges, as coupon holders in which small pieces of pipe materials can be held to study the effect of various pipe materials on biofilm growth and the resulting impact on monochloramine residual maintenance.
